# Jaw tremor: a manifestation of vascular parkinsonism? - a case report

**DOI:** 10.1186/s12883-018-1093-5

**Published:** 2018-06-30

**Authors:** Thilini B. Udagedara, Inuka Kishara Gooneratne

**Affiliations:** 1 Kegalle District General Hospital, Kegalle, Sri Lanka; 2 National Hospital of Sri Lanka, Colombo, Sri Lanka

**Keywords:** Jaw tremors, Vascular parkinsonism, Unilateral lenticular infarction

## Abstract

**Background:**

Vascular Parkinsonism **(VP)** is a heterogeneous group of conditions that manifest clinically in parkinsonian features, but are presumably of vascular cause. It is usually bilateral, non-tremulous, and frequently associated with pyramidal signs. Classically VP is described as lower body parkinsonism affecting predominantly the legs.

**Case presentation:**

A 67 years old lady presented with a history of acute onset jaw tremor, with tremor predominantly in both upper limbs. Neurological examination revealed hypomimia of the face with cogwheel rigidity and bradykinesia bilaterally, predominantly in the upper limbs without pyramidal signs. She had a marked tremor of the jaw at rest. When she was asked to open her mouth the tremor was re-emergent. Non contrast CT scan of her brain revealed an infarction in the region of putamen on the left with no evidence of diffuse subcortical white matter ischemia or extension to the caudate nucleus. She was treated with levodopa and responded well to medication.

**Conclusions:**

This case describes atypical clinical features which could be associated with VP including jaw tremor. This case also stresses the importance of initiating a trial of levodopa as certain patients may respond well to medication.

**Electronic supplementary material:**

The online version of this article (10.1186/s12883-018-1093-5) contains supplementary material, which is available to authorized users.

## Background

Parkinsonism is clinically defined by the presence of akinesia/ bradykinesia, plus one of the following signs: 4–6 Hz resting tremor, extrapyramidal rigidity and postural instability not owing to other causes [1]. “Vascular parkinsonism (VP)” is a form of atypical parkinsonism in which the parkinsonian features are of vascular cause rather than a neuro-degenerative process as in typical “Parkinson’s disease (PD)”. It accounts for 4.4–12% of all cases of Parkinsonism [2]. The following case describes atypical clinical features including jaw tremor which could be associated with VP.

## Case presentation

A 67 years old lady came to the out-patient department with a history of acute onset jaw tremor, with tremor predominantly in both upper limbs approximately 2 months prior to consultation. Her symptoms had progressed over a period of 24–48 h and remained static until the consultation. She did not have features of non-motor symptoms to suggest a diagnosis of idiopathic PD.

There was no history of previous stroke or vascular risk factors for stroke. She had not been on any medication which could cause extra-pyramidal symptoms.

General physical examination was normal. Neurological examination revealed hypomimia of the face with cogwheel rigidity and bradykinesia bilaterally (right more than left), predominantly in the upper limbs without pyramidal signs (the Unified Parkinson’s Disease Rating Scale (UPDRS) Part III; item 18–32 was 36). She had a marked tremor of the jaw at rest (Additional file 1). When she was asked to open her mouth the tremor was re-emergent (Additional file 2). There were no pyramidal signs. The rest of the neurological examination was normal which included cognition, speech, cerebellar function and bladder function.


**Additional file 1: Video 1.** “Re-emergent tremor” of the jaw. (WMV 1309 kb)



**Additional file 2: Video 2.** Upper limb dominant asymmetric extrapyramidal signs. Bradykinesia noted predominantly on the right, demonstrating fatiguing. (WMV 4556 kb)


**Fig. 1:**
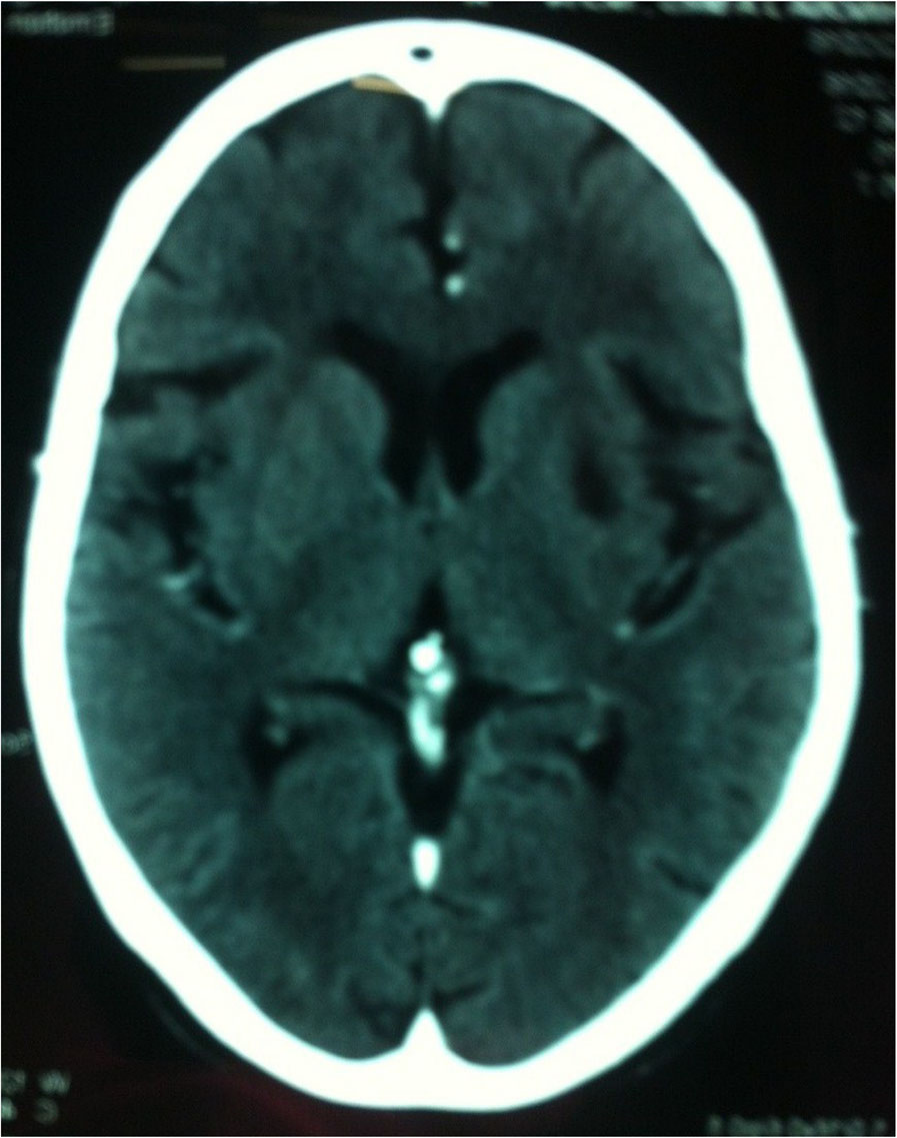
Non-contrast CT scan of the brain demonstrating an infarction in the region of the putamen on the left

Non-contrast CT scan of her brain revealed an infarction in the region of the putamen on the left with no evidence of diffuse subcortical white matter ischemia or extension to the caudate nucleus (Fig. 1). A vascular screen for stroke risk factors was negative. A DAT scan was unavailable due to lack of resources and financial constraints.

She was treated with a trial of levodopa (300 mg per day) and anti-platelet therapy which resulted in marked reduction in her jaw tremor and other extrapyramidal symptoms after 1 month of follow-up (UPDRS part III improved from 36 to 24).

## Discussion and conclusions

The patient described presented with parkinsonian features of bradykinesia, rigidity, mild rest tremor and predominant jaw tremor which were acute in onset accompanied by imaging evidence of unilateral basal ganglia stroke raising the possibility of vascular parkinsonism (VP). Zijlmans et al., [2] proposed possible criteria for the clinical diagnosis of VP and they are as follows: (a) parkinsonism, defined as bradykinesia, and at least one of the following: rest tremor, rigidity or postural instability; (b) cerebrovascular disease, defined as evidence of relevant cerebrovascular disease by brain imaging or the presence of focal signs or symptoms consistent with stroke; (c) a relationship between (a) and (b). The above patient fulfills these criteria.

VP manifests clinically with features of parkinsonism, which could be due to a single vascular cause (suggested by history), as in this case, or due to multiple strokes [3]. Classically VP is described as lower body parkinsonism affecting predominantly the legs with broad-based, shuffling, and often freezing gait and postural instability [4]. It is usually bilateral, non-tremulous, and frequently associated with pyramidal signs, pseudo-bulbar palsy, incontinence, dementia, diabetes, and hypertension [5]. The coexistence of lower body parkinsonism and cerebrovascular disease on imaging is suggestive of VP. However the clinical features in this case was of upper body predominance which can be seen in 0–4%, compared to lower body predominance seen in 60–73.7% [6].

VP has been associated with unilateral or bilateral infarcts in the striatum, lentiform nucleus, or pons [3]. Two forms of VP have been described: one with acute onset, associated with basal ganglionic infarcts as in this case, and another with insidious onset, associated with more chronic and diffuse subcortical white matter ischaemia and involving the striatum, lentiform nucleus, or pons [7, 8]. The second form often produces clinical features resembling the classical lower body parkinsonism and has a more relentless rather than stepwise progression. Infarctions affecting basal ganglia lacunae, including the thalamus, GPe and putamen that extend into the caudate and internal capsule, can mimic features of idiopathic PD [9, 10]. Infarction involving the putamen without such extension was seen in our patient. Often unilateral infarcts produce contralateral features of parkinsonism [10] and for this reason, the features of bilateral parkinsonism was unique to our case. However the extra-pyramidal symptoms were more marked on the right consistent with a left sided putaminal lesion. We postulate that the left-sided lesion, a new onset lesion was responsible for the more marked symptoms on the contra-lateral side and an older lesion on the right which is not apparent on CT imaging could have contributed to symptoms on the left. It is worth noting that unilateral putaminal infarcts often give rise to contralateral dystonia while pallidal infarcts give rise to behavioural and cognitive deficits [11]. None were features in this case.

Facial tremor has a broad differential which includes essential tremor, multiple system atrophy, drug-induced tremor or parkinsonism, or hereditary geniospasm [12]. Jaw tremor in this patient was present at rest while the mouth remained closed and was re-emergent when asked to open the mouth. Jaw tremor may occur in either essential tremor (ET) or PD, although in ET it is more typically a postural or kinetic tremor rather than a rest tremor. It has been proposed that a dominant central generator is responsible for the development of tremor in either orofacial structures or extremities in parkinsonism [13]. Data from functional neuroimaging indicates that dopaminergic dysfunction in the pallidum triggers the onset of tremor [14]. The infarct seen in our patient may have extended to involve the pallidum. The “Re-emergent tremor” is a postural tremor that appears after some delay while maintaining a posture seen in PD [15]. Facial tremors as initial manifestations of PD are uncommon [12] and are poorly described in VP. However facial tremors have been described in other forms of parkinsonism including multisystem atrophy and encephalitis lethargica [16].

The parkinsonian symptoms in this case could be due to the vascular lesion disrupting the interconnecting fibre tracts between the basal ganglia, the thalamus, and the motor cortex that leads to disruption not only of sensory-motor integration, but also of descending reticular pathways to the major centers of the brain stem [17].

Our patient showed a good response to levodopa. Although up to a half of patients with VP improve with levodopa, a robust response would favor the diagnosis of idiopathic PD. Patients with vascular lesions which either involve or are in close proximity to the nigrostriatal pathway and rare cases with abnormal DAT SPECT imaging are more likely to improve with levodopa [2]. Unfortunately due to the lack availability of such a scan we were unable to perform a DAT SPECT.

An observational clinical trial involving 17 patients with VP by Zijlmans et al. reported an excellent response to L-dopa treatment in 3 patients (mean dose 450 mg/day, range 100–1000 mg/day) while a good response was seen in 9, and moderate improvement in 2 patients during the first year of starting treatment. Three patients showed no response to L-dopa [18]. It was observed that such a positive response was seen in patients with lesions in or near the nigrostriatal pathway (ie:- macroscopically visible lacunar infarcts or lacunae caused by enlarged perivascular spaces in the putamen, caudate nucleus, and globus pallidus, or microscopic substantia nigra cell loss). Zijlmans et al. postulated that the positive response to L-dopa in VP patients was due to the remaining striatal dopaminergic nerve terminals (in a dysfunctional nigrostriatal pathway) being adequate to convert exogenous L-dopa into dopamine thus overcoming the dysfunctional thalamocortical drive. The absence of L-dopa response in certain patients with a nigrostriatal lesion may have been because of the inability of the basal ganglia to adequately increase output by L-dopa to compensate for the dysfunctional thalamocortical drive. In a recent meta-analysis which included 14 cross-sectional studies, 2 case–control studies, 2 cohort studies and 2 clinicopathological studies (17 studies were used in the analysis) it was concluded that the calculated event rate of levodopa response (odds ratio for positive response to levodopa) in VP subjects was 0.304 [95% confidence interval (CI) of 0.230–0.388], thus having a low response rate to levodopa [19]. The analysis revealed that approximately 30% of VP subjects respond to levodopa therapy. The overall odds ratio for good response to levodopa in VP with lesion in the nigrostriatal pathway vs. no lesion in the nigrostriatal pathway was 15.15 (95% CI: 5.2–44.17) concluding good response to levodopa therapy.

Often, dopaminergic therapy has no effect on Parkinsonism in most cases with bilateral putaminal lesions in which post-synaptic dysfunction is prominent. There might have been some presynaptic dysfunction due to microscopic substantia nigra cell loss in our patient which is not seen with conventional imaging and seen in histological analysis as was demonstrated in the study by Zijlmans et al., [2] Furthermore levodopa response was also reported by Hatano et al. in a patient with bilateral putaminal hemorrhages [20].

It was noted that the facial tremors in this patient was responsive to a dose of L-dopa of 300 mg per day. A study showed that response of facial tremor to an acute levodopa challenge showed high sensitivity, specificity, positive and negative likelihood ratios for the diagnosis of PD [12]. The efficacy of levodopa on facial/ jaw tremor in VP has not been described. Considering the above it should be considered that the presence of cerebrovascular disease, being a frequent incidental finding in older idiopathic PD patients, the stroke in this patient could have unmasked and aggravated her already existing parkinsonian symptoms. However our patient did not complain of non-motor symptoms which could have suggested a diagnosis of idiopathic PD.

The presence of acute onset jaw tremor and its dramatic response to levodopa in the presence of unilateral lentiform infarction in this case illustrates the heterogeneity of clinical presentation of VP. The possibility of idiopathic PD being unmasked due to acute basal ganglia stroke also needs to be considered in this situation.

## Abbreviations

ET: Essential tremor
GPe: External globus pallidus
PD: Parkinson Disease
VP: Vascular parkinsonism
